# Nitrogen regulation of fungal secondary metabolism in fungi

**DOI:** 10.3389/fmicb.2014.00656

**Published:** 2014-11-28

**Authors:** Bettina Tudzynski

**Affiliations:** Institute of Biology and Biotechnology of Plants, Westfaelische Wilhelms-University MuensterMuenster, Germany

**Keywords:** nitrogen regulation, secondary metabolites, AreA, AreB, MeaB, Nmr1, GS

## Abstract

Fungi occupy diverse environments where they are constantly challenged by stressors such as extreme pH, temperature, UV exposure, and nutrient deprivation. Nitrogen is an essential requirement for growth, and the ability to metabolize a wide variety of nitrogen sources enables fungi to colonize different environmental niches and survive nutrient limitations. Favored nitrogen sources, particularly ammonium and glutamine, are used preferentially, while the expression of genes required for the use of various secondary nitrogen sources is subject to a regulatory mechanism called *nitrogen metabolite repression*. Studies on gene regulation in response to nitrogen availability were carried out first in *Saccharomyces cerevisiae*, *Aspergillus nidulans*, and *Neurospora crassa*. These studies revealed that fungi respond to changes in nitrogen availability with physiological and morphological alterations and activation of differentiation processes. In all fungal species studied, the major GATA transcription factor AreA and its co-repressor Nmr are central players of the nitrogen regulatory network. In addition to growth and development, the quality and quantity of nitrogen also affects the formation of a broad range of secondary metabolites (SMs). Recent studies, mainly on species of the genus *Fusarium*, revealed that AreA does not only regulate a large set of nitrogen catabolic genes, but can also be involved in regulating production of SMs. Furthermore, several other regulators, e.g., a second GATA transcription factor, AreB, that was proposed to negatively control nitrogen catabolic genes by competing with AreA for binding to GATA elements, was shown to act as activator of some nitrogen-repressed as well as nitrogen-induced SM gene clusters. This review highlights our latest understanding of canonical (AreA-dependent) and non-canonical nitrogen regulation mechanisms by which fungi may regulate biosynthesis of certain SMs in response to nitrogen availability.

## INTRODUCTION

It is well known that secondary metabolites (SMs) are not required for viability but provide a competitive advantage to the microorganism producing them in various ways. They may improve nutrient availability (e.g., in the form of chelating agents such as siderophores), protect it against environmental stresses (e.g., pigments against UV irradiation), enhance its competitive interactions for nutrients with other microorganisms in ecological niches, decrease the fitness of their hosts, e.g., plants, animals, or humans, and act as a metabolic defense mechanism (Breitling et al., 2013). Many SMs act as pathogenicity factors, such as the host selective T-toxin from *Cochliobolus heterostrophus* (Turgeon and Baker, 2007), the cyclic peptide AM-toxin from *Alternaria alternata* (Markham and Hille, 2001) or the trichothecene deoxynivalenol (DON) produced by *Gibberella zeae* (Proctor et al., 1995; Jansen et al., 2005).

Secondary metabolites are only produced during specific conditions, and their biosynthesis is subject to diverse regulatory controls. As a consequence, many of the SM biosynthetic genes show little or no expression under typical laboratory conditions, and therefore the potential new SMs are either not produced, or are present at levels that are too low to be detected by standard methods (Brakhage, 2013). The increasing number of sequenced fungal genomes has revealed that fungi probably produce many more SMs than originally expected, though most of these new SMs are only predicted by bioinformatics analysis of putative SM gene clusters.

In the pre-genomics era, culture supernatants were screened to identify new SMs with activities of interest. These classical methods for activating SM genes involved the manipulation of culture conditions exemplified by the OSMAC (one strain, many compounds) approach (Bode et al., 2002; Craney et al., 2013). This simple and inexpensive method led to the discovery of many new metabolites and gave first insight into the complex regulatory network which includes widely conserved general, as well as pathway-specific, regulation principles.

Also by limiting the rate of availability of a single nutrient, e.g., in continuous fermentations, normally silent gene clusters can be activated by controlling the specific growth rate. The strength of this approach has been demonstrated by growing *Aspergillus nidulans* in a chemostat under nitrogen limitation. By this approach, two novel polyketide metabolites, sanghaspirodins A and B, were discovered (Scherlach et al., 2011). Orsellinic acid biosynthesis is also induced under nitrogen limitation in continuous culture and is suggested to be a precursor for sanghaspirodins biosynthesis (Scherlach et al., 2011; **Table 1**). These studies revealed a significant impact of nutritional factors, such as carbon and nitrogen sources, on SM production and morphological differentiation (Keller et al., 1997; Calvo et al., 2002).

**Table 1:** Nitrogen-regulated secondary metabolites and regulators involved.

Secondary metabolite	Fungus	Nitrogen regulation	Nitrogen regulators involved	Reference
Aflatoxin	*Aspergillus parasiticus Aspergillus flavus*	NO_3_ repression, NH_4_ induction	AreA binds AflR promoter	Feng and Leonard (1998), Chang et al. (2000), Ehrlich and Cotty (2002)
Alternariol	*Alternaria alternata*	Nitrogen repression	No data	Brzonkalik et al. (2011)
Apicidin F	*F. fujikuroi*	Nitrogen induction	AreA-independent; AreB-dependent	Niehaus et al. (2014b)
Beauvericin	*F. oxysporum*	Nitrogen induction	AreA-dependent	López-Berges et al. (2014)
Bikaverin	*F. fujikuroi*	Nitrogen repression	AreA not essential; GS, MeaB, MepB, and TOR involved	Teichert et al. (2004, 2006, 2008), Wiemann et al. (2009), Wagner et al. (2010, 2013)
Cephalosporin	*Acremonium chrysogenum*	Nitrogen repression	AreA-dependent	Li et al. (2013)
Carotenoids	*F. fujikuroi*	Nitrogen repression	No data	Rodriguez-Ortiz et al. (2009)
Fumonisin	*F. verticillioides*	Nitrogen repression	AreA-dependent	Kim and Woloshuk (2008)
Fumonisin	*F. proliferatum*	Nitrogen repression	Nitrogen starvation stress regulated by HOG MAP kinase	Kohut et al. (2009)
Fusaric acid	*F. fujikuroi*	Nitrogen induction	AreA-independent; AreB-dependent	Niehaus et al. (2014a)
Fusarielin H	*F. graminearum*	Nitrogen repression	AreA-dependent	Giese et al. (2013)
Fusarin C	*F. fujikuroi*	Nitrogen induction	AreA and AreB-independent; GS-dependent	Díaz-Sánchez et al. (2012), Niehaus et al. (2013)
Fusarubins	*F. fujikuroi*	Nitrogen repression; alkaline pH	No data	Studt et al. (2012, 2013a)
Gibberellins	*F. fujikuroi*	Nitrogen repression	AreA-and AreB-dependent; MepB, GS, TOR, and MeaB involved	Mihlan et al. (2003), Teichert et al. (2004, 2006, 2008), Schönig et al. (2008), Wagner et al. (2010, 2013), Michielse et al. (2014)
Ochratoxin	*Aspergillus ochraceus*, *P. verrucosum*	Nitrogen-induced	No data	Abbas et al. (2009)
Orsellinic acid	*Aspergillus nidulans*	Nitrogen starvation-induced	No data	Scherlach et al. (2011)
Patulin	*P. urticae*	Nitrogen repression	No data	Rollins and Gaucher (1994)
Penicillin	*P. chrysogenum*	Nitrogen repression	Nre binds to the *ACV-IPN*-promoter	Haas and Marzluf (1995)
Spiroanthrones	*Aspergillus nidulans*	Nitrogen starvation-induced	No data	Scherlach et al. (2011)
Sterigmatocystin	*Aspergillus nidulans*	NH_4_ repression NO_3_ induces	No data	Feng and Leonard (1998)
Trichothecenes (DON)	*F. graminearum*	Nitrogen repression	AreA-dependent	Min et al. (2012), Giese et al. (2013)
Zearalenone	*F. graminearum*	Nitrogen repression	Contradictory results to AreA-dependency	Min et al. (2012), Giese et al. (2013)

## REGULATION OF SECONDARY METABOLISM BY NITROGEN AVAILABILITY

Of all environmental factors, the quality and quantity of the nitrogen source used in the growth media have a special effect not only on growth and differentiation, but also on the biosynthesis of many known fungal SMs, e.g., production of sterigmatocystin and aflatoxin in different *Aspergillus* species (Chang et al., 1995; Feng and Leonard, 1998; Calvo et al., 2002; Ehrlich and Cotty, 2002), gibberellin (GA; Jefferys, 1970; Giordano and Domenech, 1999), fusarubin (Studt et al., 2012), bikaverin (Wiemann et al., 2009), fusaric acid (Niehaus et al., 2014a), and fusarin (Díaz-Sánchez et al., 2012; Niehaus et al., 2013) in *Fusarium fujikuroi*, fumonisin in *F. verticillioides* (Kim and Woloshuk, 2008), or cephalosporin, penicillin, and patulin in *Acremonium chrysogenum*, *Penicillium chrysogenum*, and *P. urticae*, respectively (Rollins and Gaucher, 1994; Haas and Marzluf, 1995; Li et al., 2013). An overview of fungal SMs whose biosynthesis is affected by nitrogen availability is given in **Table 1**.

Recent genome-wide microarray experiments under nitrogen-sufficient and nitrogen-limiting conditions in *F. fujikuroi* revealed that the expression of 30 out of the 45 putative SM gene clusters depends on the quantity and quality of the nitrogen source. Among them are 13 clusters with a polyketide synthase (PKS) gene (e.g., those for fusaric acid, bikaverin, fusarubin, and fumonisin), the two with a diterpene cyclase (DTC) gene (GA and carotenoid clusters), two with a sesquiterpene cyclase (STC) gene, 11 with a non-ribosomal peptide synthetase (NRPS) gene (e.g., the apicidin F cluster), one with a dimethylallyl tryptophan synthase (DMATS) gene, and one with the only type III-PKS gene (Wiemann et al., 2013).

However, for most of the nitrogen-regulated SMs in fungi the molecular mechanism of the nitrogen dependency is not well understood. To better understand the complex nitrogen regulation network in general, the following paragraph provides an overview of fungal nitrogen regulators which ensure the preferential use of primary nitrogen sources and also confer the ability to use different secondary nitrogen sources when appropriate.

## NITROGEN REGULATION IN FUNGI: THE GATA-TYPE TRANSCRIPTION FACTORS AreA AND AreB

Filamentous fungi are able to use many compounds as sole nitrogen sources, but preferentially use energetically favored nitrogen sources such as NH_4_^+^ and glutamine for as long as they are present in the medium. In the absence of these sources, less easily assimilated nitrogen sources such as nitrate, urea, uric acid, amines, amides, purines, and pyrimidines may also be used (Marzluf, 1997; Wong et al., 2008). The regulatory mechanism that enables preferential utilization of easily assimilated nitrogen sources in one circumstance, but selective utilization of these secondary nitrogen sources in another circumstance, is called *nitrogen metabolite repression*. This global regulatory circuit ensures the transcriptional activation of structural genes encoding enzymes and permeases required for scavenging and degradation of energetically less favored nitrogen sources (Wiame et al., 1985; Marzluf, 1997; Fraser et al., 2001; Magasanik and Kaiser, 2002). In ascomycetes, nitrogen metabolite repression is mediated by transcription factors belonging to the GATA family. The key regulatory genes *areA* in *A. nidulans* and *nit-2* in *N. crassa* were cloned more than 25 years ago (Fu and Marzluf, 1987; Caddick, 1992). The predicted amino acid sequences of AreA and NIT2 revealed a high level of similarity, mainly in the DNA-binding domain consisting of a Cys_2_/Cys_2_-type zinc finger motif and the adjacent basic region, while the N-terminal region is highly variable and dispensable for function in *A. nidulans* (Fu and Marzluf, 1990; Kudla et al., 1990; Caddick and Arst, 1998). Cross-genus complementation showed that *nit-2* and *areA* are functional orthologs (Davis and Hynes, 1987). NIT2/AreA were found to preferentially bind to at least two 5′HGATAR DNA motifs located within 30 bp of each other (Ravagnani et al., 1997).

The standard model of nitrogen metabolite repression is that AreA/NIT2 and their orthologs in several other ascomycetes (reviewed in Wiemann and Tudzynski, 2013) mediate de-repression of many genes involved in utilization of secondary nitrogen sources in the absence of glutamine and ammonium. However, the fungus activates the transcription of catabolic genes only when their substrates are available. For this substrate-specific gene activation, additional pathway-specific transcription factors are involved which mediate induction of a set of genes in response to a specific inducer. The best characterized example is the nitrate assimilation system in *A. nidulans* where AreA and the pathway-specific transcription factor NirA act synergistically and physically interact to assure the use of nitrate as sole nitrogen source (Narendja et al., 2002; Muro-Pastor et al., 2004; Berger et al., 2008). Interpretation of recent data has unraveled the individual contributions of NirA and AreA in this complex activation/inactivation process. It was shown that AreA is required for histone H3 acetylation and concomitant chromatin restructuring in the bidirectional promoter of the nitrate and nitrite reductase genes, *niaD*, and *niiA* (Berger et al., 2008). NirA also participates in the chromatin-opening process during nitrate induction, but by an H3 acetylation-independent mechanism and only requires the presence of nitrate. In nitrogen-starved cells, when elevated AreA chromatin occupancy and histone H3 hyperacetylation have been obtained, the chromatin remodeling function of NirA is dispensable. However, continuous presence of high nitrate assimilation and subsequent accumulation of intracellular glutamine (Schinko et al., 2010) lead to lowered AreA activities. Under these conditions, the interaction between the nitrate-activated NirA and the NplA/KapK nuclear export complex seems to be disrupted and results in nuclear retention of NirA and partial compensation for reduced AreA occupancy at the promoter (Bernreiter et al., 2007; Berger et al., 2008).

The activity of AreA itself is regulated by several signaling processes that report the extracellular nitrogen availability and the intracellular nitrogen status (Caddick et al., 2006). Under nitrogen-limiting conditions, AreA activity is partially de-repressed due to increased levels of *areA* transcription and *areA* mRNA stability compared with nitrogen-sufficient conditions (Langdon et al., 1995; Platt et al., 1996; Morozov et al., 2000, 2001). Furthermore, AreA activity is controlled by nitrogen starvation-induced nuclear translocation of AreA and subsequent elevated AreA-dependent gene expression in *A. nidulans* and *F. fujikuroi* (Todd et al., 2005; Michielse et al., 2014). In *F. graminearum* AreA accumulates in the nucleus under both nitrogen-limiting conditions and with nitrate as a sole nitrogen source. GFP-AreA was still visible in the nuclei in rich complete medium though with much lower intensity of GFP fluorescence *(*Min et al., 2012).

In *A. nidulans*, AreA contains six conserved nuclear localization sites (NLSs), but only the simultaneous mutation of all of them prevented AreA nuclear accumulation (Hunter et al., 2014). In addition to nuclear translocation, the export from the nucleus also seems to be an additional mechanism for regulation of AreA activity: upon addition of a rich nitrogen source, AreA is exported from the nucleus within minutes and can no longer activate expression of its target genes (Todd et al., 2005).

In some plant pathogenic fungi, such as *F. oxysporum*, and the opportunistic human pathogen *P. marneffei*, the AreA orthologs have been found to be required for full virulence, probably due to the failure of mutants to fully adapt to nitrogen-poor conditions during infection (Divon et al., 2006; Bugeja et al., 2012). In *P. marneffei* it was assumed that AreA is likely to contribute to its pathogenicity by also regulating the expression of potential virulence factors such as extracellular proteases (Bugeja et al., 2012).

Another level of regulation of AreA activity involves its interaction with the co-repressor NmrA/Nmr1 in *A. nidulans*, *N. crassa*, and *F. fujikuroi* (Fu et al., 1988; Xiao et al., 1995; Andrianopoulos et al., 1998; Schönig et al., 2008). It has been proposed that AreA and NIT2 transcriptional activity is inhibited by interaction with NmrA and Nmr1, respectively. In *N. crassa* it has been shown that both the DNA-binding domain (residues 732–821) and the 12-amino-acid carboxy-terminal tail (residues 1006–1036) of NIT2 are essential for interaction with Nmr1. The level of de-repression achieved with the NIT2 proteins mutated in either the zinc finger or the C-terminus was similar to that observed in complete loss-of-function *nmr1* mutants (Pan et al., 1997). In *A. nidulans*, deletion of the 12 C-terminal residues of AreA or mutations in the zinc finger region also lead to a partially de-repressed phenotype similar to that of the Δ*nmrA* mutant (Platt et al., 1996).

Furthermore, proteolytic degradation of NmrA in *A. nidulans* occurred in an ordered manner, preferentially at the C-terminal site, thereby preventing the binding to the AreA zinc finger (Zhao et al., 2010). These data reveal a potential new layer of control of nitrogen metabolite repression by the ordered proteolytic cleavage of NmrA.

It has been reported that *nmrA* expression in *A. nidulans* is high under conditions of nitrogen-sufficiency, opposite to the pattern of *areA* expression (Wong et al., 2007). However, this is not the case in *F. fujikuroi* where *nmr1* is an AreA target gene whose expression is strictly repressed in the presence of adequate nitrogen (Schönig et al., 2008; Wagner et al., 2010). Despite this different expression pattern, Nmr1 in *F. fujikuroi* interacts with AreA as in *N. crassa* and *A. nidulans*, and deletion and over-expression of *nmr1* resulted in high sensitivity and resistance towards chlorate, respectively, indicating its regulating effect on nitrate reductase activity by determining AreA activity (Schönig et al., 2008).

In *A. nidulans*, full activation of some AreA-dependent genes, e.g., *gdhA*, requires also the function of a Zn(II)2Cys6 zinc binuclear cluster transcription factor, TamA, a homolog of the *S. cerevisiae* Dal81p protein (Davis et al., 1996). Recently it has been shown, that TamA has dual functions as a DNA-binding transcription factor and a non-DNA-binding co-activator of AreA which interacts with the C-terminal residues of AreA (Davis et al., 1996; Downes et al., 2013, 2014). At present the role of TamA has not been characterized in any other fungus.

In contrast to *S. cerevisiae*, which involves four GATA-type transcription factors in the regulation of nitrogen use, the positively acting Gln3p and Gat1p, and the negatively acting Dal80p (Uga43p) and Gzf3p (Nil2p; reviewed in Magasanik and Kaiser, 2002) filamentous ascomycetes have only two GATA-type nitrogen regulators, namely AreA and AreB. While AreA/NIT2 are responsible for activation of genes that allow utilization of energetically less-favored nitrogen sources in a similar manner to the yeast homologs Gln3p and Gat1p, AreB was suggested to repress the same set of genes as it has been shown for the yeast GATA factors Dal80p and Gzf3p (Wong et al., 2009). The first functional analyses of the second GATA transcription factor, AreB/NreB, in *A. nidulans* and *P. chrysogenum*, respectively, confirmed its proposed role as repressor of AreA target genes, probably through DNA-binding competition. Over-expression of AreB and NreB in these fungi resulted in loss of AreA/NreA-dependent gene expression indicating that this GATA factor negatively modulates AreA/NreA activity (Haas et al., 1997; Wong et al., 2009).

Recently it has been shown that in *F. fujikuroi* AreB can act both as repressor and activator of nitrogen-regulated gene expression. Thus, both GATA factors are essential for expression of the GA biosynthetic genes (see below). Some other AreA-dependent genes, such as *mepC* (Teichert et al., 2008) and *nmr1* (Schönig et al., 2008), showed significantly elevated transcription in the Δ*areB* deletion mutant indicating that AreA and AreB have common sets of target genes but can affect their expression in opposite ways. However, it is not yet known whether AreB directly binds to the promoters of AreA target genes, or if its effect is due to down-regulation of *areB* transcript levels in the *ΔareA* mutant (Michielse et al., 2014).

In *A. nidulans*, detailed analysis of *areA* and *areB* single and double mutants has shown that both GATA transcription factors negatively regulate the expression of arginine catabolism genes *agaA* and *otaA* under nitrogen-repressing conditions. AreA is necessary for the ammonium repression of *agaA* and *otaA* under conditions of carbon repression, while AreB is involved under carbon-limiting conditions (Macios et al., 2012).

Interestingly, no impact on the expression of nitrogen-regulated genes has been shown for the AreB ortholog in *N. crassa*, Asd4. This transcription factor plays a role in development of asci and ascospores (Feng et al., 2000).

In *A. nidulans* and *F. fujikuroi*, the *areB* gene encodes multiple products all containing the GATA zinc finger and a leucine zipper motif (Conlon et al., 2001; Michielse et al., 2014).

However, the biological role of these different proteins is not well understood. Recently, it has been shown for the first time by a bimolecular fluorescence complementation (BiFC) approach that one of the AreB isoforms in *F. fujikuroi* interacts with AreA in the nucleus when starved of nitrogen (Michielse et al., 2014). Furthermore, expression of both *areA* and *areB* is repressed by nitrogen and induced by nitrogen starvation. Interestingly, the *areB* transcription level is decreased 6-fold in the Δ*areA* mutant when nitrogen is scarce, whilst AreB does not affect *areA* expression.

## OTHER REGULATORS INVOLVED IN NITROGEN REGULATION OF METABOLIC PROCESSES IN FILAMENTOUS FUNGI

How do fungi sense the extracellular nitrogen availability and their intracellular nitrogen status? Furthermore, it is not understood clearly whether fungi can sense different types of nitrogen sources, or whether the intracellular pool of *glutamine* alone provides the stimulus for expression or repression of nitrogen-responsive genes.

**FIGURE 1:**
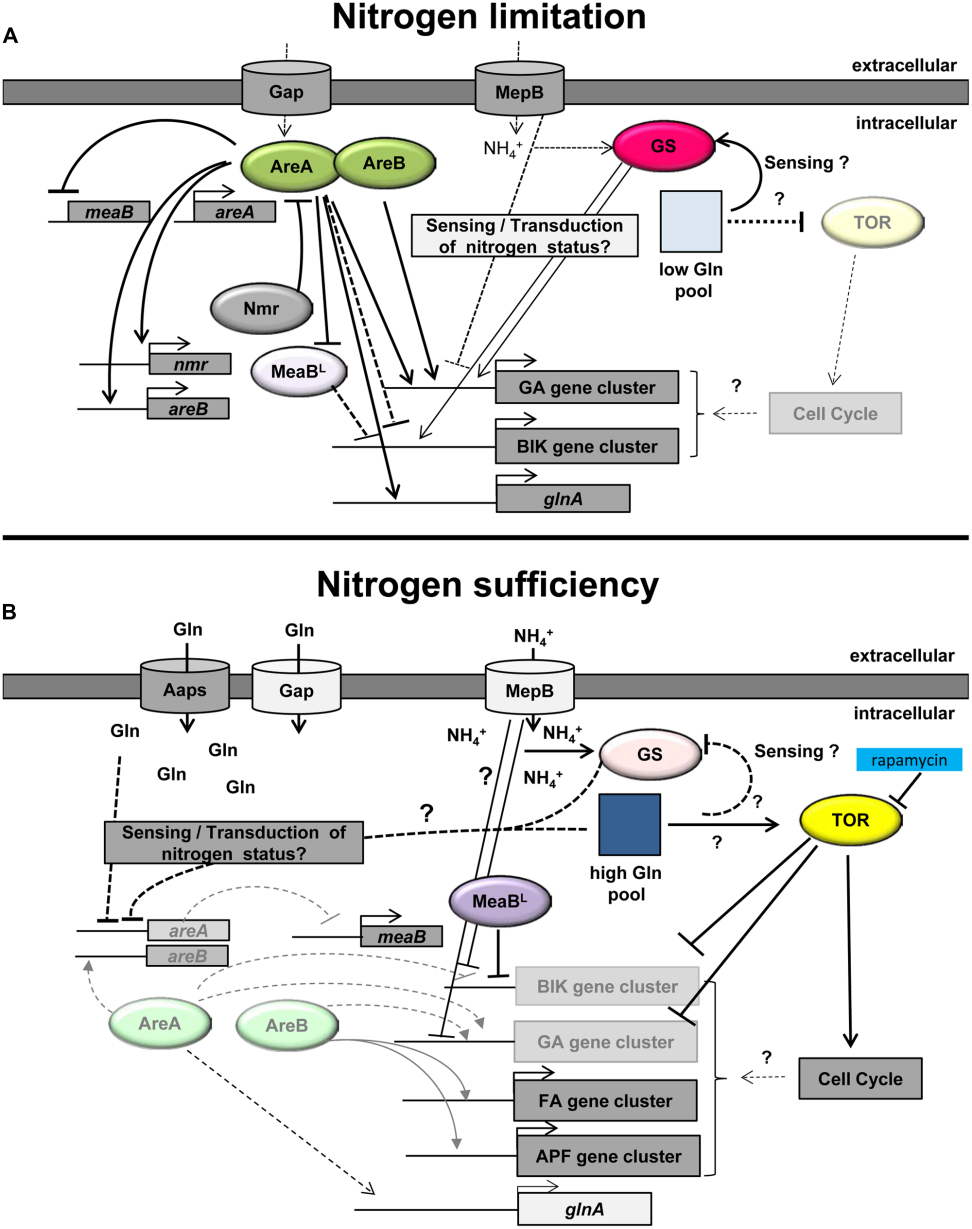
**Model of the nitrogen regulation of secondary metabolism in *Fusarium fujikuroi*. (A)** Under nitrogen-limiting conditions, the intracellular glutamine (Gln) pool is low (light square). This nitrogen status of the cell is probably sensed by the GS and/or additional nitrogen sensors, e.g., the ammonium permease MepB. Under these conditions, the activity of TOR is low and *areA* is highly expressed. AreA subsequently activates the expression of *areB*. Both GATA transcription factors, AreA and AreB, are essential for the activation of the GA and probably also the fumonisin gene clusters. Active AreA represses the transcription of the negatively acting bZIP transcription factor MeaB^L^ resulting in high expression of the AreA-independent bikaverin gene cluster. AreA also induces the expression of a variety of genes including *nmr*, *glnA*, and *mepB*. Increasing levels of Nmr interact with AreA thereby putatively modifying its activity by a feed-back loop. **(B)** Under nitrogen sufficient conditions, TOR is active resulting in activation of the cell cycle. Rapamycin inhibits TOR activity resulting in partial derepression of nitrogen-repressed SM genes. The high intracellular Gln pool (dark blue square) is putatively sensed by the GS and/or other sensors and results in low *areA* mRNA levels. The AreA-dependent ammonium permease MepB and the general amino acid permease (Gap) are only slowly expressed, but other amino acid permeases (AAPs) and substrate-specific nitrogen transporters facilitate transport of nitrogen into the cell. The decreased level of active AreA leads to reduced GA (and fumonisin) gene expression and induction of *meaB*^L^ expression. MeaB^L^ itself is involved in repression of bikaverin (BIK) gene expression. The remaining levels of AreB directly or indirectly activate the expression of the nitrogen-induced fusaric acid (FA) and apicidin F (APF) gene clusters (modified after Wiemann and Tudzynski, 2013).

These processes have been intensely studied in *S. cerevisiae* where the “TOR” (target of rapamycin) kinase and the downstream TOR signaling pathway play a crucial role as global regulators of cell growth. Under nitrogen-sufficient conditions, TOR is able to sense amino acid-derived signals and to stimulate a set of anabolic processes, including translation, transcription, and ribosome biogenesis, whereas nitrogen depletion or inhibition of TOR by the TOR inhibitor rapamycin triggers G1 cell cycle arrest, protein synthesis inhibition, and autophagy (Rohde and Cardenas, 2004; Rubio-Texeira, 2007; Rohde et al., 2008; **Figure 1**).

However, the mechanisms by which signals of nutrient availability are sensed by TOR and transduced to transcription factors to elicit a response are not fully understood. In *S. cerevisiae*, TOR regulates two highly conserved phosphatases, Sit4, and PP2A, which in turn regulate cellular localization of the two positively acting GATA-type transcription factors, Gln3 and Gat1. In the presence of sufficient nitrogen, Gln3 and Gat1 are cytoplasmic, and NCR genes are not expressed. However, nitrogen starvation or treatment with rapamycin result in immediate nuclear translocation of both transcription factors and subsequent transcription of nitrogen-repressed genes (Tate et al., 2010).

Much less is known on the role of the TOR kinase in filamentous fungi. Despite the presence of putative homologs of the TOR kinase and the other components of the TOR cascade in the sequenced genomes of filamentous fungi (Fitzgibbon et al., 2005; Teichert et al., 2006), there is currently no experimental evidence that TOR signaling acts through regulating subcellular localization of the GATA factors AreA and AreB. Genome-wide transcriptome analysis in *F. fujikuroi* with RNA from rapamycin-treated mycelium revealed a partial de-repression of some AreA target genes. Like in yeast, genes involved in ribosome biogenesis, and translation initiation/elongation are down-regulated by rapamycin, whereas genes involved in autophagy and protection against oxidative stress are up-regulated by the TOR inhibitor (Teichert et al., 2006). In the tomato pathogen *F. oxysporum*, ammonium-mediated repression of cellophane penetration is reversed by rapamycin, indicating that this negative regulation of penetration by ammonium also requires an active TOR cascade (López-Berges et al., 2010). The nitrogen source and TOR also control other virulence-related functions, such as vegetative hyphal fusion and root adhesion in *F. oxysporum* (López-Berges et al., 2010).

Assimilation of different nitrogen sources, such as nitrate, nitrite, urea, or amino acids, results in formation of NH_4_^+^ which is subsequently converted first to glutamate via glutamate dehydrogenase (GdhA), and then to glutamine via *glutamine synthetase* (GS). It became the widely accepted view that glutamine – and not ammonium, nitrate or glutamate – is the key effector of nitrogen metabolite repression to ensure the preferential utilization of reduced nitrogen sources such as ammonium and glutamine when multiple N resources are available (Dunn-Coleman and Garrett, 1980; Dunn-Coleman et al., 1981; Wiame et al., 1985; Caddick et al., 1994; Magasanik and Kaiser, 2002).

Recent studies in *A. nidulans* showed a 50% drop in glutamine concentration within 5 min after transferring the mycelium into nitrogen-free medium, while the levels of most of the other amino acids were not significantly different from the levels measured in ammonium-grown cultures. These data suggest that glutamine is indeed the marker for the nitrogen status (Berger et al., 2008). The question to be asked is whether glutamine alone is the key co-repressor of many metabolic processes as proposed by Premakumar et al. (1979) and Margelis et al. (2001). It has been proposed for a long time that the GS has an important role not only in providing glutamine, but also as key regulator in the nitrogen regulatory network in yeast and filamentous fungi. Indeed, in *N. crassa* (Dunn-Coleman and Garrett, 1980; Dunn-Coleman et al., 1981; Mora, 1990) and *S. cerevisiae* (Crespo et al., 2002; Magasanik, 2005), treatment with methionine sulfoximine (MSX), a specific inhibitor of the GS, or loss-of-function mutations in the GS, lead to increased expression of nitrogen-regulated genes in cells grown on a preferred nitrogen source. In *F. oxysporum*, GS activity is strictly required for ammonium-mediated inhibition of Cellophane penetration, suggesting that glutamine, rather than ammonium, acts as a signal for nitrogen repression (López-Berges et al., 2010).

A regulatory role for GS has been also postulated for *F. fujikuroi.* A macroarray approach comparing transcriptional profiles of wild-type *F. fujikuroi* with those of the *gln1* deletion strain revealed a set of genes that are strongly up- or down-regulated in the mutant. For example, the gene for the cross pathway control, *CPC1*, and genes involved in translation control (*eEF1α*; *eIH5a*), stress response (*ddr48*; *cipC*), ribosome biogenesis, and histone modification (histone acetyltransferases) all indicate that GS plays a major role in regulating different processes in the fungal cells (Teichert et al., 2004; Wagner et al., 2013). Furthermore, point mutations in an ammonium binding domain of the *F. fujikuroi* GS led to de-repression of several nitrogen-repressed genes with ammonium as nitrogen source. These data indicate that the putative NH_4_^+^ binding site D60/S62 causes *loss of NH_4_^+^ sensing* and subsequent loss of wiring of the repressing signal towards gene expression of nitrogen-regulated genes (Wagner et al., 2013; **Table 2**). Interestingly, deletion of the GS-encoding gene *gln1* resulted in significant up-regulation of several nitrogen regulatory genes, such as *areA*, *areB*, *mepB*, and *nmr*, strongly supporting the hypothesis that the GS itself plays a regulatory role (Wagner et al., 2013).

**Table 2:** Functions of regulators involved in nitrogen-dependent control of secondary metabolism.

Regulator	Function	Reference
**AreA**	GATA transcription factor; positive regulator of GA, fumonisin, DON, zearalenone, fusarielin H, beauvericin, and cephalosporin gene expression AreA is involved in chromatin accessibility and is essential for full virulence of some plant pathogens	Tudzynski et al. (1999), Berger et al. (2008), Kim and Woloshuk (2008), Min et al. (2012), Giese et al. (2013), Li et al. (2013), López-Berges et al. (2014)
**AreB**	GATA transcription factor, positive regulator of GA, fusaric acid and apicidin F biosynthesis AreA interacts with AreB under nitrogen-limiting conditions	Michielse et al. (2014), Niehaus et al. (2014a,b)
**Nmr**	Negative regulator of AreA activity as the deletion of *nmr1* results in increased chlorate sensitivity However, deletion of *nmr1* does not overrule the repression of GA, DON, zearalenone and fusarielin H genes under high nitrogen conditions in *Fusarium* spp.;	Schönig et al. (2008), Giese et al. (2013)
**Tor**	Inhibition of Tor by rapamycin led to up-regulation of GA and bikaverin biosynthesis genes under nitrogen-limiting conditions, but does not overrule their repression at high nitrogen	Teichert et al. (2006)
**MepB**	Strong de-repression of the GA and bikaverin biosynthetic genes in the *mepB* deletion mutant under nitrogen-sufficient conditions; sensing or regulatory role of MepB suggested	Teichert et al. (2008), Shnaiderman et al. (2013)
**GS**	Deletion of *gln1* resulted in loss of GA, bikaverin, fusarin and apicidin F gene expression and up-regulation of *areA*, *areB*, *mepB*, and *nmr1* genes; Postulated sensing and regulatory roles of the GS; affects energy metabolism	Teichert et al. (2004), Niehaus et al. (2013, 2014b), Wagner et al. (2013)
**MeaB**	bZIP transcription factor; affects expression of several nitrogen-regulated genes; elevated expression of GA and bikaverin biosynthesis genes in the deletion mutant under nitrogen-limiting conditions; overexpression of *A. nidulans meaB* led to impaired *in planta* aflatoxin B1 production	López-Berges et al. (2010), Wagner et al. (2010), Amaike et al. (2013)
**Vel1/VeA**	FfVel1 may partially overcome nitrogen repression of bikaverin genes *veA* mutant in *F. oxysporum* is impaired in growth on nitrate	Wiemann et al. (2010), López-Berges et al. (2014)

Besides the TOR pathway and the GS, one of the three ammonium permeases, “MepB,” was shown to have transport and sensing functions and is probably part of the nitrogen regulation network (Teichert et al., 2008). Deletion of *mepB* resulted in impaired growth on media with low concentrations of ammonium as well as in de-repression of multiple genes (e.g., the genes *MTD1* and *AAP8* encoding a peptide transporter and an amino acid permease) that are normally repressed at high concentrations of ammonium (Teichert et al., 2008).

In *Colletotrichum gloeosporioides*, ammonia uptake by the germinating spores of the wild-type, but not of the Δ*mepB* strain with compromised ammonium transport, activated cAMP-mediated transcription of regulatory and catalytic PKA subunits (Shnaiderman et al., 2013). As a consequence, Δ*mepB* mutants showed 75% fewer appressoria and colonization than the wild-type demonstrating that MepB contributes to the virulence of this fungus, probably by activating the cAMP pathway similar to Mep2p, the sensing ammonium permease (“transceptor”) in yeast (Van Nuland et al., 2006).

An additional central component of the nitrogen regulation network in fungi is the cross-pathway transcription factor “Cpc1” mediating the transcriptional up-regulation of genes involved in amino acid metabolism under conditions of amino acid imbalance (Barthelmess and Kolanus, 1990; Tian et al., 2007). In *F. fujikuroi* it has been shown that the expression of *cpc1* and Cpc1 target genes is not activated under nitrogen-limiting conditions. However, the *cpc1* gene as well as several genes involved in amino acid biosynthesis are significantly up-regulated in the *F. fujikuroi gln1* deletion mutant due to the significantly decreased glutamine pool and subsequent amino acid imbalance (Teichert et al., 2004; Schönig et al., 2009). Interestingly, in *A. nidulans*, a binding sequence motif for Cpc1 is present in the *prnB* promoter that is also regulated by AreA (Tazebay et al., 1997). Elucidation of the mechanisms by which AreA is interconnected with Cpc1 and GS should lead to a major advance in understanding the role of AreA in amino acid metabolism.

Beside these nitrogen regulators, the bZIP transcription factor “MeaB” has been shown to be involved in nitrogen repression of a set of genes. The *meaB* gene in *A. nidulans* was identified by mutations conferring resistance to toxic amino acid analogs and methylammonium ions, resulting in de-repression of nitrogen-regulated genes (Arst and Cove, 1973; Polley and Caddick, 1996; Wong et al., 2007). However, it is not clear whether there is a functional or physical interaction of MeaB with AreA. Contradictory reports exist about the role of MeaB in regulation of *nmrA* expression in *A. nidulans*. Whereas Wong et al. (2007) reported that under conditions of nitrogen sufficiency MeaB activates *nmrA* expression by binding to a conserved sequence in the *nmrA* promoter, only a slight or no impact of MeaB on *nmrA*/*nmr1* transcription was shown in comparative studies on *A. nidulans* and *F. fujikuroi*, respectively (Wagner et al., 2010). In addition, the postulated binding site for MeaB in the *A. nidulans nmrA* promoter is not conserved in the promoters of *nmrA* homologs in other genera of filamentous fungi (Wagner et al., 2010). Despite these different reports on the impact of MeaB on *nmrA*/*nmr1* expression, both MeaB, and NmrA/Nmr1 were regarded as repressive nitrogen regulatory proteins in *N. crassa*, *Fusarium* spp., and *Aspergillus* spp. (Pan et al., 1997; Wong et al., 2007; López-Berges et al., 2010; Wagner et al., 2010; Amaike et al., 2013). In *F. oxysporum*, MeaB and TOR were shown to be required for repression of the ability to penetrate Cellophane films overlaid on agar plates with high concentrations of ammonium. Both inhibition of TOR by its inhibitor rapamycin and deletion of *meaB* consistently enhanced the ability to penetrate Cellophane in the presence of ammonium (López-Berges et al., 2010). In *F. fujikuroi*, several AreA target genes, such as *mtd1* and *aap1* encoding a peptide permease and an amino acid permease, respectively, are partially up-regulated under starvation conditions in the Δ*meaB* mutant. However, these genes are not de-repressed under nitrogen sufficient conditions, when MeaB is translocated to the nucleus. Therefore, the de-repressing effect seems to be indirect (Wagner et al., 2010).

## THE ROLE OF THE GATA FACTORS AreA AND AreB IN REGULATING SECONDARY METABOLISM

For a long time the major nitrogen regulator AreA and its orthologs were thought to be involved exclusively in regulating the use and metabolization of non-favored nitrogen sources (Arst and Cove, 1973; Marzluf, 1997). AreA orthologs have been identified and deleted in a number of filamentous ascomycetes (Wiemann and Tudzynski, 2013), but the impact of AreA inactivation on secondary metabolism is still not well understood.

The first indication that AreA ortologs may regulate SM biosynthesis came from studies on NreA in *P. chrysogenum* (Haas and Marzluf, 1995). The authors showed that this AreA ortholog binds not only to the intergenic promoter regions of the nitrate and nitrite reductase genes (*niiA-niaD*), but also to the intergenic region between *acvA* and *pcbC*, encoding the first two enzymes in penicillin biosynthesis. Unfortunately, the NreA-encoding gene has not been deleted due to very low homologous integration rates in this fungus. Therefore, the direct proof of an involvement of this GATA factor in regulation of penicillin biosynthesis has still not been achieved. Furthermore, it has been suggested that AreA may play a role in the regulation of the aflatoxin gene cluster in *Aspergillus parasiticus*, but this also remains to be proved (Chang et al., 2000).

In *F. fujikuroi*, it has been known for more than 50 years that fermentation of *F. fujikuroi* for GA production delivers highest yields under nitrogen-limiting conditions, and that the red pigment bikaverin, a by-product of GA fermentation, is induced under the same conditions (Borrow et al., 1964; Jefferys, 1970; Bu’lock et al., 1974). However, studies on the molecular mechanism of this nitrogen regulation were possible only after the identification of GA and bikaverin biosynthesis genes (Tudzynski and Hölter, 1998; Linnemannstöns et al., 2002; Wiemann et al., 2009). GAs were the first SMs for which the essential role of the GATA-type transcription factor AreA were proved unequivocally (**Table 1**; **Figure 1A**). Deletion of AreA almost fully abolished GA biosynthesis and expression of GA cluster genes (Tudzynski et al., 1999; Mihlan et al., 2003). Furthermore, AreA was shown to directly bind to the GATA/TATC sequence elements in the promoters of the GA genes by gel mobility shift assays. The binding of AreA was analyzed in more detail using the promoter of the *ent*-kaurene oxidase (*P450-4*) gene fused to the *Escherichia coli uidA* reporter gene (Mihlan et al., 2003). These findings were unexpected because GAs have no nitrogen in their structure and therefore cannot serve as nitrogen sources for the fungus.

As mentioned above, AreA activity was shown to be negatively regulated under nitrogen sufficient conditions by interacting with the repressor protein NmrA/Nmr1 in *A. nidulans* and *F. fujikuroi* (Lamb et al., 2003, 2004; Schönig et al., 2008). While the consequences of *nmrA* deletion on secondary metabolism have not been studied in *A. nidulans*, deletion of *nmr1* in *F. fujikuroi* did not result in the expected significant up-regulation of GA gene expression (Schönig et al., 2008). However, the Δ*nmr1* mutant is highly sensitive to chlorate indicating that Nmr1 specifically interacts with AreA to prevent nitrate utilization in the presence of glutamine, but does not act as a general repressor of AreA in all AreA-dependent pathways (Schönig et al., 2008).

More recently, some SMs from different *Fusarium* species were shown to be nitrogen repressed in an AreA-dependent manner. In *F. verticillioides*, the *areA* deletion strain was incapable of producing fumonisins on mature maize kernels and was compromised in its ability to grow under these *in planta* conditions unless ammonium was added. Furthermore, expression of *fum1, fum8,* and *fum12* was abolished under inducing *in vitro* conditions, while a mutant that constitutively expresses *areA* was able to produce fumonisins even under repressing conditions. These data indicate that AreA is essential for *FUM* gene expression (Kim and Woloshuk, 2008; Picot et al., 2010; **Tables 1** and **2**).

Recently, AreA was functionally characterized in *F. graminearum*, a pathogen of wheat and other cereals (Min et al., 2012; Giese et al., 2013). The virulence of Δ*areA* strains on wheat heads was significantly reduced compared with the wild-type strain. While Min et al. (2012) revealed loss of trichothecene biosynthesis, but no effect on zearalenone biosynthesis in the Δ*areA* strain, Giese et al. (2013) described AreA as a global regulator which is required for the production of DON, zearalenone, and fusarielin H regardless of the nutrient medium (**Tables 1** and **2**). However, the drastically decreased product levels and the presence of several putative tandem AreA binding sites, especially in the promoters of the zearalenone gene cluster, is not conclusive evidence for direct binding of AreA to the toxin gene promoters. The fusarin C and aurofusarin biosynthetic genes contained similar numbers of putative AreA binding sites, although these SMs are not affected in the Δ*areA* mutant.

Deletion of the putative AreA repressor gene *nmr* in *F. graminearum* had little effect on either growth or toxin production. Generally the *nmr* deletion mutant produced very low levels of DON, zearalenone, and fusarielin H under nitrogen sufficient conditions equivalent to the wild-type strain (Giese et al., 2013; **Table 2**). Similarly, GA production in *F. fujikuroi* was still repressed by high nitrogen in the *nmr1* deletion mutant although Nmr1 was shown to interact with AreA and to affect nitrate reductase activity (Schönig et al., 2008).

*In F. oxysporum*, AreA also contributes to chromatin accessibility and expression of a Velvet-regulated NRPS gene cluster, responsible for the biosynthesis of the mycotoxin beauvericin (López-Berges et al., 2014). Transcript levels of the key enzyme-encoding gene *BeaS* was significantly reduced in both the Δ*areA* and the Δ*veA* mutant. The authors suggest a combinatorial role for the Velvet complex and AreA in nitrogen use and secondary metabolism because the *F. oxysporum* Δ*veA* mutant was impaired in growth on nitrate (López-Berges et al., 2014).

Recently, the involvement of AreA in cephalosporin production has been demonstrated in *Acremonium chrysogenum* (Li et al., 2013). Consistent with the reduction of cephalosporin production, the transcription of *pcbAB*, *cefD2*, *cefEF*, and *cefG* encoding the enzymes for cephalosporin production was reduced in the Δ*AcareA* mutant. Band shift assays showed that AcAreA bound not only to the bidirectional promoter of the nitrate/nitrite reductase-encoding genes *niaD* and *niiA*, but also to the bidirectional promoter region of *pcbAB*-*pcbC*. Sequence analysis showed that all the AcAreA binding sites contain the consensus GATA elements. These results indicated that AcAreA plays an important role both in the regulation of nitrogen metabolism and cephalosporin production in *Acremonium chrysogenum* (Li et al., 2013; **Table 1**).

Surprisingly, AreA is not essential for the expression of bikaverin genes (*bik1*–*bik6*) in *F. fujikuroi* although they are co-regulated with GA genes under nitrogen-limiting conditions. Unexpectedly, *bik1* (former *pks4*) expression and bikaverin production were even stronger in the Δ*areA* mutant compared to the wild-type (Linnemannstöns et al., 2002). Therefore, a second non-canonical, AreA-independent mechanism of nitrogen metabolite repression must exist that mediates repression of *bik* genes at high nitrogen concentrations (Wiemann et al., 2009; Wiemann and Tudzynski, 2013). Furthermore, the loss of the VeA-encoding genes *Ffvel1* or *FfareA* affected nitrogen-mediated GA and bikaverin synthesis differently. Expression of the GA biosynthetic genes was significantly down-regulated in both the *Ffvel1* and *areA* deletion mutants compared with the wild-type, while the expression of the bikaverin cluster genes *bik1-3* were significantly stronger in the Δ*Ffvel1* mutant compared with the wild-type and the *areA* deletion strain and was still detected in the Δ*Ffvel1* mutant under normally repressing high nitrogen (60 mM glutamine) conditions (Wiemann et al., 2010). These data indicate that FfVel1 may partially overcome nitrogen repression of bikaverin genes.

Recently, a second family of PKS-derived red pigments has been identified in *F. fujikuroi*, the fusarubins (Studt et al., 2012). These pigments are responsible for the coloration of the perithecia. The fusarubin biosynthetic genes (*fsr1*–*fsr6*) are repressed by high nitrogen similarly to the *bik* genes. However, in contrast to the *bik* genes, which need both low nitrogen and acidic pH, the *fsr* genes are only expressed under low nitrogen conditions and alkaline pH (Studt et al., 2012). To keep alkalinity constant during the whole cultivation time in liquid media, nitrate has to be used, a nitrogen source which cannot be used by the Δ*areA* mutant. Therefore, a potential role of AreA in nitrogen-repressed fusarubin gene expression and biosynthesis cannot be excluded (Studt et al., 2012).

As for bikaverin and fusarubins, the expression of the structural genes of the carotenoid pathway, *carRA* and *carB*, is transiently increased upon nitrogen removal (**Table 1**). However, an involvement of AreA in this regulation has not been shown (Rodriguez-Ortiz et al., 2009).

A milestone in understanding the role of the two GATA transcription factors AreA and AreB were two important findings: (1) that both AreA and AreB can be involved in regulating expression of SM genes and (2) that AreB does not generally act as a negative counterpart of AreA (Michielse et al., 2014).

So far, a role of AreB as positive regulator of some SMs has been described only in *F. fujikuroi*. First of all, both AreA and AreB were shown to be essential for expression of the GA biosynthesis genes and concomitant GA production under conditions of nitrogen starvation. Both AreA and AreB co-localized and interact with each other in the nucleus under GA production conditions as shown by use of BiFC (Michielse et al., 2014; **Table 2**).

Interestingly, AreB is not only involved in regulating expression of nitrogen starvation-induced SMs. Recently it has been demonstrated that fusaric acid and apicidin F, both induced under nitrogen sufficient conditions, are not produced and the biosynthetic genes are almost not expressed in the Δ*areB* mutant (Niehaus et al., 2014a,b; **Table 1**; **Figure 1B**). However, AreB is not involved in regulation of all nitrogen-induced SMs as the formation of fusarin C is not affected in the Δ*areB* mutant of *F. fujikuroi* (Niehaus et al., 2013).

## THE INVOLVEMENT OF OTHER NITROGEN REGULATORS IN SECONDARY METABOLISM

In *A. nidulans* and *N. crassa*, “NmrA/Nmr1” have been postulated to play a major role in regulating AreA activity. However, no data exist on its potential repressing effect on SM production. In *F. fujikuroi*, deletion of *nmr1* resulted in high sensitivity to chlorate indicating that Nmr1 affects the activity of nitrate reductase via its interaction with Are (Mihlan et al., 2003; Wagner et al., 2010). However, deletion of *nmr1* did not result in de-repression of the AreA-dependent GA biosynthesis genes in the presence of ammonium or glutamine, and over-expression of *nmr1* revealed only a slight repression (**Table 2**). The limited impact of *nmr1* deletion on the expression of AreA-dependent SM genes on the one hand, and the AreA-independent expression of other nitrogen-regulated SM genes, e.g., those for bikaverin, aurofusarin, and fusarin biosynthesis (Wiemann et al., 2009; Giese et al., 2013), suggest that additional factors must be involved in nitrogen regulation in general, and particularly in regulation of SM.

In *A. nidulans*, *F. fujikuroi* and *F. oxysporum*, the bZIP transcription factor “MeaB” affects expression of several nitrogen-regulated genes (López-Berges et al., 2010; Wagner et al., 2010; Amaike et al., 2013). However, not much is known about the role of MeaB in regulating SM. In *F. fujikuroi*, deletion of the gene resulted in increased transcription of the GA and bikaverin biosynthesis genes under inducing low nitrogen conditions, but did not overcome repression by glutamine. It is notable that full de-repression of the AreA-independent bikaverin biosynthesis genes was observed in the Δ*meaB/*Δ*areA* double mutant suggesting that both AreA and MeaB act as repressors of bikaverin biosynthesis in different pathways. Studies are still needed to test whether this strong de-repressing effect is due to direct binding of one or the other TF, or if both regulators act indirectly by affecting the activity of other regulators involved in bikaverin biosynthesis, e.g., the pathway-specific Zn(II)2Cys6 transcription factor Bik5 (Wagner et al., 2010).

In *A. nidulans*, loss of *meaB* did not significantly affect secondary metabolism. However, the *OE::meaB* strain had a much stronger phenotype. The mutant is impaired in seed colonization, lipase activity and *in planta* aflatoxin B1 production (Amaike et al., 2013). Further experiments will allow an unambiguous integration of MeaB into the established nitrogen regulation network in filamentous fungi.

Studies in *F. fujikuroi* and *Colletotrichum gloeosporioides* revealed that the ammonium permease “MepB” might be involved in sensing external nitrogen availability and the intracellular nitrogen status of the cell (Teichert et al., 2008; Shnaiderman et al., 2013). However, an impact of MepB on SM has been shown so far only for *F. fujikuroi*. The deletion of *mepB* in this fungus resulted in strong de-repression of the GA and bikaverin biosynthetic genes at early stages of growth when ammonium is still present at sufficient levels and secondary metabolism is not switched on in the wild-type strain (**Figure 1**). To be sure that this de-regulation of SM production is not due to reduced ammonium transport capacity, but to a sensing defect, the gene *mepC* encoding the second high capacity transporter MepC, was over-expressed in the Δ*mepB* background. While the strong growth defect was partially overcome, the SMs were still de-repressed at high ammonium concentrations, supporting the suggestion that MepB plays a sensing or regulatory role in addition to its function as a permease (Teichert et al., 2008). However, downstream signaling components, as in *C. gloeosporioides* (Shnaiderman et al., 2013), have not yet been identified in *Fusarium* spp..

As mentioned before, the “GS” seems to play an important role as key regulator in the nitrogen regulation network, mainly by regulating the intracellular glutamine levels (Dunn-Coleman and Garrett, 1980; Dunn-Coleman et al., 1981; Mora, 1990; Crespo et al., 2002; Magasanik, 2005). However, its potential function in regulating SM production has been studied only recently. In *F. fujikuroi*, deletion of the GS-encoding gene *gln1* resulted in surprising loss of GA and bikaverin production instead of the expected increased product yields due to the decreased glutamine pool in the mutant (Teichert et al., 2004). The GA and bikaverin biosynthetic genes are significantly down-regulated even under nitrogen-limiting conditions. The same results were obtained by inhibiting GS by MSX (Teichert et al., 2004). Recently, the biosynthesis of two nitrogen-induced metabolites, fusarin C (Niehaus et al., 2013) and apicidin F (Niehaus et al., 2014b) was also shown to be absolutely dependent on a functional GS. Therefore, the *gln1* mutant can neither produce GAs and bikaverin, nor fusarin and apicidin F (**Figure 1**).

Interestingly, the two GS-encoding genes of *N. crassa* (*Ncgln1* and *Ncgln2*) and even those of *Streptomyces coelicolor, glnA*, and *glnII,* fully restored not only the wild type-like growth, but also SM gene expression and product formation of the *F. fujikuroi gln1* deletion strain indicating the high level of functional conservation between prokaryotes and eukaryotes (Wagner et al., 2013).

Complementing the Δ*gln1* mutant with 14 different *F. fujikuroi gln1* copies with site-directed mutations in the 14 highly conserved GS domains resulted either in restoration of both glutamine synthesis and SM production, or of none of these activities. However, three site-directed mutations in the *gln1* gene partially restored secondary metabolism and GS-dependent gene expression, but not glutamine formation, demonstrating for the first time that the catalytic and regulatory roles of GS can be separated. One of these three mutants with a mutation in a postulated ammonium binding domain (mutant D60A/S62A) revealed a partial de-repression of the AreA-dependent GA and AreA-independent bikaverin gene expression when grown with NH_4_^+^ as sole nitrogen source (Wagner et al., 2013).

The reason for the strong impact of the GS on SM gene expression is unclear. However, the significantly reduced growth of the Δ*gln1* mutant (only 15% dry weight compared to the wild-type) in media with high glucose and low amounts of glutamine compared to the wild-type suggests a defect in efficient glucose use and ATP generation. All complemented *gln1* mutant strains with functional GS proteins revealed not only restored SM but also growth similar to the wild-type strain, while the glutamine auxotrophic strains accumulated significantly less biomass similar to the Δ*gln1* mutant. Surprisingly, the three deregulated mutants (D60A/S62A, G246A/G248A, and S72A/D73A) accumulated five- to sixfold more dry weight than the Δ*gln1* mutant and produce SMs. These data indicate that the down-regulation of secondary metabolism in the Δ*gln1* mutant is due to suboptimal energy balance and carbon source availability (Wagner et al., 2013).

In addition to transmembrane localized permeases which have transport and sensing functions (“transceptors”), and the GS which seems to be involved in ammonium sensing, additional internal nitrogen sensors must exist which are able to determine the nitrogen status of the cell and to affect expression of nitrogen-repressed SMs. One of them is the “TOR” protein kinase in *F. fujikuroi*. Genome-wide transcriptional analysis of the wild-type treated or not treated with rapamycin revealed a similar set of TOR-controlled genes, which are involved in anabolic processes, including translation, transcription, and ribosome biogenesis, similarly to the rapamycin-sensitive genes found in *S. cerevisiae* (Rohde and Cardenas, 2004; Teichert et al., 2006). In contrast to yeast which does not produce any SMs, inhibition of TOR by rapamycin resulted in partial deregulation of GA and bikaverin biosynthesis genes, but also of some other genes subject to nitrogen metabolite repression. However, this partial deregulation was obtained only with nitrogen concentrations no higher than 10 mM (Teichert et al., 2006). Rapamycin was insufficient to override the repressing effect of high nitrogen concentrations. Currently, it is not known whether inhibition of TOR by rapamycin results in nuclear translocations of the GATA transcription factors AreA and/or AreB, and if Sit4- and PP2A-like phosphatases are involved in dephosphorylation of AreA as it has been shown for the AreA homolog Gln3p in *S. cerevisiae* (Georis et al., 2008; Tate and Cooper, 2013). In *F. oxysporum* it has been shown that rapamycin treatment increased transcript levels of three AreA target genes (*niaD*, *niiA*, and *mepB*; López-Berges et al., 2010). The up-regulation of the sensing ammonium permease *mepB* might also contribute to the elevated expression of GA and bikaverin biosynthetic genes upon rapamycin treatment in *F. fujikuroi*.

Besides the TOR cascade, other signaling components seem to be involved in nitrogen sensing and subsequent regulation of secondary metabolism. In *F. proliferatum* it has been shown that the “HOG (high osmolarity glycerol)-type MAPK” pathway senses nitrogen starvation stress and regulates adaptation to these new conditions. Deletion of the HOG-encoding gene resulted in much stronger elevation of *FUM* gene expression upon transfer into a nitrogen-free medium compared to the wild-type but had no impact on FUM gene expression under nitrogen sufficient conditions (Kohut et al., 2009). A role of the stress-activated MAP kinase Sak1, a homolog of Hog1, in nitrogen sensing, and pigment production has also been shown in *Aspergillus fumigatus* (May et al., 2005).

Another example for the role signaling cascades may play in nitrogen regulation of secondary metabolism is the Gα protein/adenylate cyclase-mediated repression of the fusarubin production in *F. fujikuroi*. These PKS-derived perithecial pigments were only produced under nitrogen-limiting alkaline conditions (5 mM NaNO_3_). Deletion of the Gα subunit FfG1 resulted in strong up-regulation of *fsr* gene expression and fusarubin production not only under low nitrate conditions but also under normally repressing high (60 mM) nitrate conditions (Studt et al., 2013a).

## NITROGEN AVAILABILITY AFFECTS THE HISTONE MODIFICATION PATTERNS

Recent studies in several fungi showed that SM gene clusters in fungi can be regulated by chromatin-modifying enzymes, such as histone acetylases and deacetylases as well as histone methylases (Williams et al., 2008; Bok et al., 2009; Strauss and Reyes-Dominguez, 2011; Soukup et al., 2012). In general, gene expression has been associated with acetylation of histone H3 lysine 9 (H3K9ac) and dimethylation of histone H3 lysine 4 (H3K4me2), whereas gene silencing has been associated with trimethylation of histone H3 lysine 9 (H3K9me3; Bannister and Kouzarides, 2011; Strauss and Reyes-Dominguez, 2011).

In *F. fujikuroi*, many putative SM biosynthetic gene clusters were mapped to individual chromosomes, and most of them were shown to be located within subtelomeric regions. These regions are often subject to regulation by posttranslational modification of histones. To show whether microarray-based expression profiles and production levels for a certain SM fit with enrichment of a specific histone mark at the cluster region, genome-wide ChIP-seq analysis under nitrogen-limiting and nitrogen sufficient conditions were performed by using antibodies specific to two activating (H3K9ac and H3K4me2) and one silencing (H3K9me3) modifications. The presence of H3K9ac was correlated with gene expression across the GA, bikaverin, and fumonisin gene clusters at low concentrations of nitrogen, while H3K9ac was almost completely absent under repressing high-nitrogen conditions (Wiemann et al., 2013). These data support the hypothesis that histone acetylation is also associated with gene transcription in *F. fujikuroi*. However, this is not always the case. For example, the fusaric acid cluster showed little enrichment for H3K9ac under the inducing high nitrogen conditions (Wiemann et al., 2013). Therefore, other factors, including global or specific transcription factors, additional histone modifications, or other external signals such as pH or plant signals can also regulate expression of cluster genes.

A link between nitrogen regulation and histone modifications has been recently shown for the bikaverin biosynthesis in *F. fujikuroi*. While the *bik* genes are only expressed under nitrogen-limiting conditions in the wild-type strain, high *bik* gene expression levels under repressing (60 mM glutamine) and low expression levels under normally inducing conditions (6 mM glutamine) have been observed in the histone deacetylase deletion strain Δ*ffhda1* (Studt et al., 2013b). This contrasting expression pattern was confirmed by Northern blot and high-performance liquid chromatography with diode-array detection (HPLC-DAD).

## CONCLUSION

Fungi are able to respond to quantitative and qualitative changes in nitroogen availability through complex regulatory mechanisms. Components of this regulatory network are nitrogen sensors, signaling cascades, e.g., the TOR cascade, transcription factors, and other regulatory proteins that might be able to interact. In contrast to *S. cerevisiae* with four GATA transcription factors involved in nitrogen regulation, filamentous fungi have only two, AreA and AreB. The former factor predominantly acts as positive regulator activating the expression of a set of nitrogen-regulated genes when preferred nitrogen sources, such as glutamine or ammonia, are limited. In the past years significant progress has been made in understanding how AreA functions. Besides its binding to the promoters of target genes, work in *A. nidulans* revealed an additional important role of AreA: the ability to mediate chromatin remodeling by increasing histone H3 acetylation. Much less is known about the role of AreB. Recent work in *F. fujikuroi* clearly showed that AreB can act both as positive and negative regulator and it regulates common target genes with AreA, but also AreB-specific target genes.

Beside genes involved in utilization and metabolization of different nitrogen sources, AreA is now well accepted as regulator of SMs. After the finding that AreA is essential for expression of GA biosynthetic genes in *F. fujikuroi*, several other SMs, mainly produced by species of the genus *Fusarium,* were also shown to depend on AreA. Recently it has been shown that both AreA and AreB are essential for GA gene expression, whereas only AreB positively affects apicidin F and fusaric acid biosynthesis.

Beside the two major transcription factors AreA and AreB, some other regulators have been shown to affect SM production, such as MeaB, Tor, and the GS. Also one of the three ammonium permeases, MepB, seems to play a role as sensor in addition to its transport function. A model of the function of individual components of the nitrogen regulation network in *F. fujikuroi* with emphasis on secondary metabolism is given in **Figure 1**.

However, despite the progress made in studying nitrogen regulation of secondary metabolism, the molecular mode of action, and possible interactions between and cross-talks with the regulators are not well understood. Because of the importance of nitrogen availability in regulating secondary metabolism, fundamental studies are needed to shed light on the functions of individual regulators, but also on the entire network, from sensing the nitrogen signal to the alteration of expression profiles.

## Conflict of Interest Statement

The author declares that the research was conducted in the absence of any commercial or financial relationships that could be construed as a potential conflict of interest.
